# Supplementary material for: Selective constraint acting on TLR2 and TLR4 genes of Japanese Rana frogs
Source: PeerJ. 2018 May 25;6:e4842. doi: 10.7717/peerj.4842 (PMC5971840; doi:10.7717/peerj.4842)

Table S1. Detailed summary of samples used for tissue collection and transcriptome library construction.

| Species | Life stage (sample/s for transcriptome data set from Lau et al 2017) | Source | Captivity information | DNA extraction* for this TLR study |
| --- | --- | --- | --- | --- |
| *R. japonica* | Adult (blood, skin, spleen)  s29 tadpole (body) | Etajima 34°16'14''N 132°28'37''E  “ “ | E  E | Skin (n = 5)  - |
| *R. ornativentris* | Adult (blood, skin, spleen)  s29 tadpole (body)  s24 tadpole (skin) | Yoshiwa 34°25'04’’N 132°05'15''E  “ “  Yokohama 35°20'21"N 139°35'09"E | T  T  E | Skin (n = 5)  -  - |
| *R. t. tagoi* | Adult (blood, skin, spleen) | Shobara 34°05'04''N 132°49'43''E | A | Skin (n = 5) |

E– raised in captivity from eggs, T- raised in captivity from tadpoles, A- adults collected in field, and held in captivity for over . *Adults were euthanized in June 2015, and samples stored in RNAlater at -20^o^C until DNA extraction in February 2017.

Table S2. Primers used to amplify TLR2 and TLR4 in all three *Rana* species, as well as primers used for sequencing.

|  | Full length size (from *Xenopus*) | Primers (5’ – 3’) | Size amplified (bp) |
| --- | --- | --- | --- |
| RanaTLR2 | 2340 bp | RanaTLR2_F: TGRTTGCATACATATGGAGTTG  RanaTLR2_R: GTGGTCCTCTGGCTGAAGAG | 2348 |
| RanaTLR4 | 2260 bp  (exon 3) | RanaTLR4_F: CTGGCAAGCCTTTCTGAACT  RanaTLR4_R: AGCGGARCATCAACTTTACG | 2072 |
| RanaTLR2 sequencing primers | - | RanaTLR2_nested_F1: TCTYAGGCAATATGCAAACG  RanaTLR2_nested_F2: TGTATAGGGAGTGTCTGCTAACTG  RanaTLR2_nested_F3: TTTGGAGCATTTGGATGTSA  RanaTLR2_ nested_F4: TGCTCCTGTCAGTTTGTGGA  RanaTLR2_ nested_R1: YGCTGCACTGTTGTCAGTTAG  RanaTLR2_ nested_R2: AAAGTCAAYGAGMCGGTTTT | - |
| RanaTLR4 sequencing primers | - | RanaTLR4_ nested_F1: ATGATGCRGTCTTGTTGTGA  RanaTLR4_ nested_F2: CTTCAAGGCTCGCTACTCC  RanaTLR4_ nested_R1: CAATGTTGGTGAGCCGTTYA  RanaTLR4_ nested_R2: GGTGTTCTGGTGTCTTGCATT | - |

Table S3. TLR2 and TLR4 genotypes determined from the 3 focal species (n = 5 individuals per species). Alleles found in more than one individual are in bold.

| Species | Animal ID | TLR2 genotype | TLR4 genotype |
| --- | --- | --- | --- |
| *R. japonica* | Rj8 | RajaTLR2_02/03 | RajaTLR4_**01**/**03** |
|  | Rj9 | RajaTLR2_04/05 | RajaTLR4_**01**/02 |
|  | RjS1 | RajaTLR2_**01** hom | RajaTLR4_**03** hom |
|  | RjS3 | RajaTLR2_**01** hom | RajaTLR4_**03**/04 |
|  | RjS4 | RajaTLR2_06/07 | RajaTLR4_**03**/05 |
| *R. ornativentris* | Ro1 | RaorTLR2_09/10 | RaorTLR4_03/**04** |
|  | Ro3 | RaorTLR2_07/08 | RaorTLR4_**02**/**04** |
|  | Ro4 | RaorTLR2_03/04 | RaorTLR4_01/**02** |
|  | Ro5 | RaorTLR2_01/02 | RaorTLR4_**04**/07 |
|  | Ro6 | RaorTLR2_05/06 | RaorTLR4_05/06 |
| *R. tagoi tagoi* | Rt1 | RataTLR2_09/10 | RataTLR4_05/06 |
|  | Rt10 | RataTLR2_07/08 | RataTLR4_01 hom |
|  | Rt11 | RataTLR2_05/06 | RataTLR4_02 hom |
|  | Rt12 | RataTLR2_03/04 | RataTLR4_07/08 |
|  | Rt13 | RataTLR2_01/02 | RataTLR4_03/04 |

Figure S1. Nucleotide alignment of representative TLR2 transcripts from the three focal Japanese *Rana* species, as well as *Lithobates catesbieanus* (transcriptome data), *Xenopus laevis* (XM_002933491) and *Nanorana parkeri* (XM_018557931). Primers are indicated by boxes. 5’ to 3’ direction of nested primers are represented by arrows.


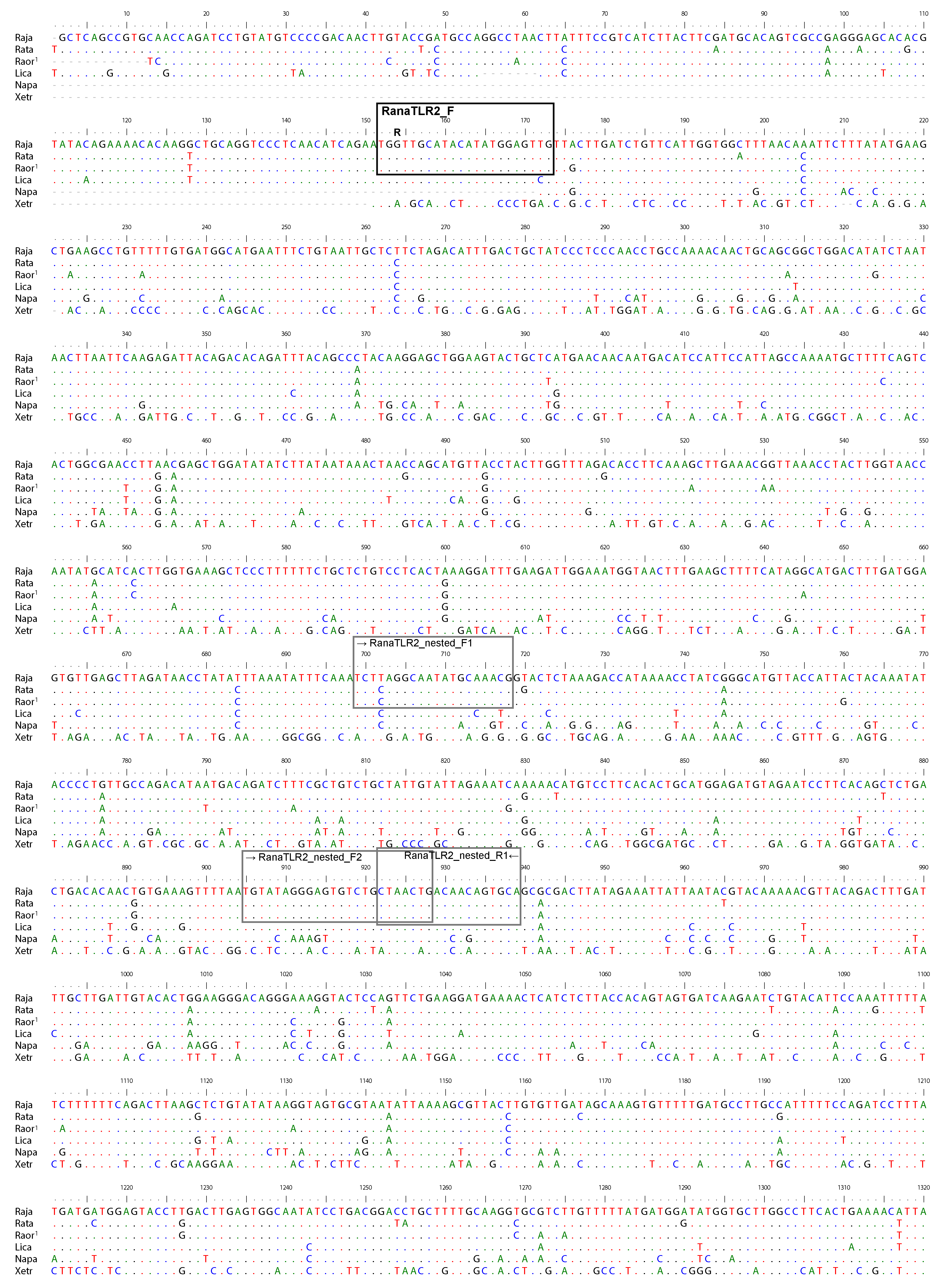

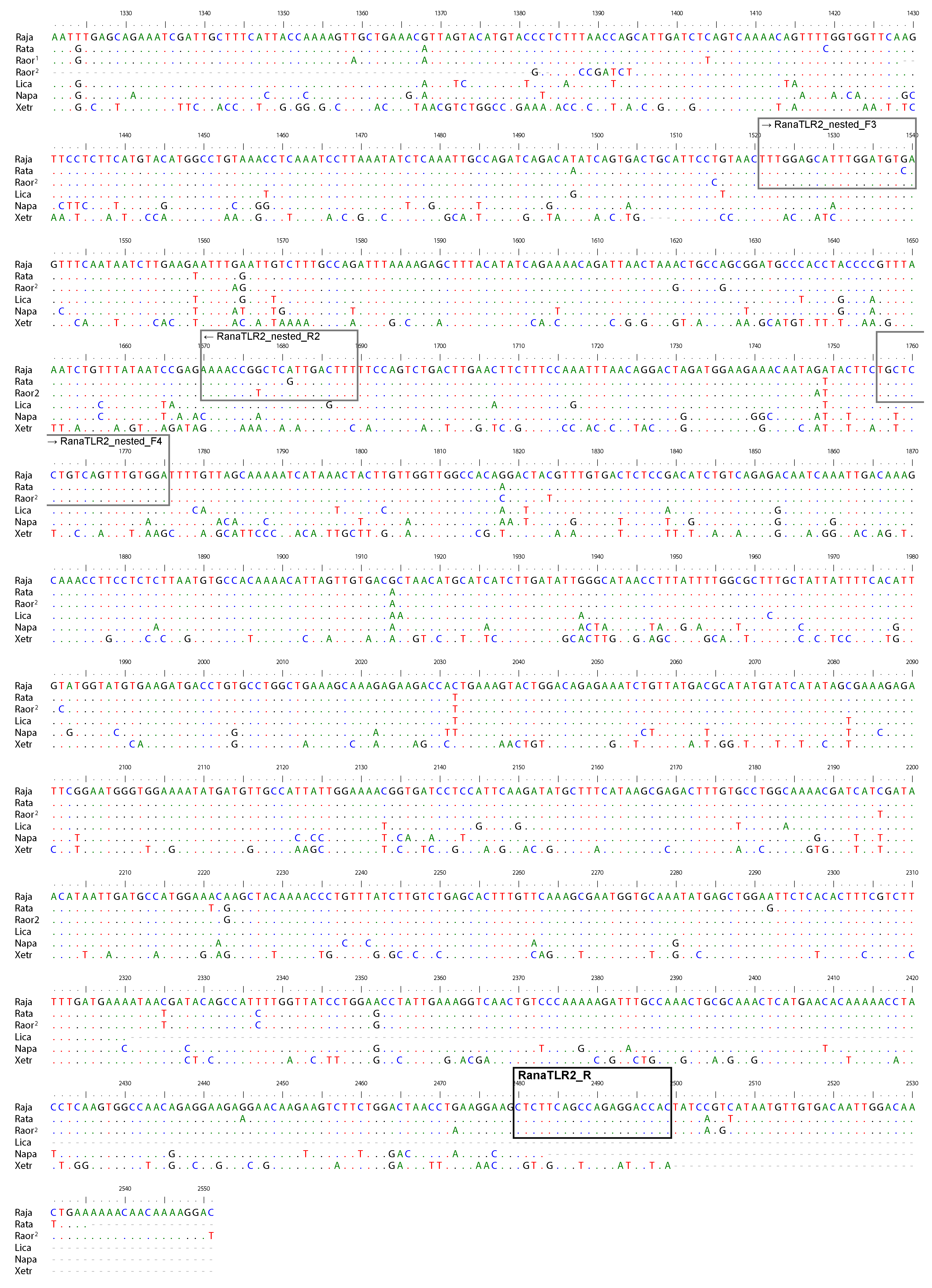


Figure S2. Nucleotide alignment of representative TLR4 transcripts from the three focal Japanese *Rana* species, as well as *Lithobates catesbieanus* (KV969108), *Xenopus laevis* (XM_018232906) and *Nanorana parkeri* (XM_018565865). Primers are indicated by boxes. 5’ to 3’ direction of nested primers are represented by arrows.


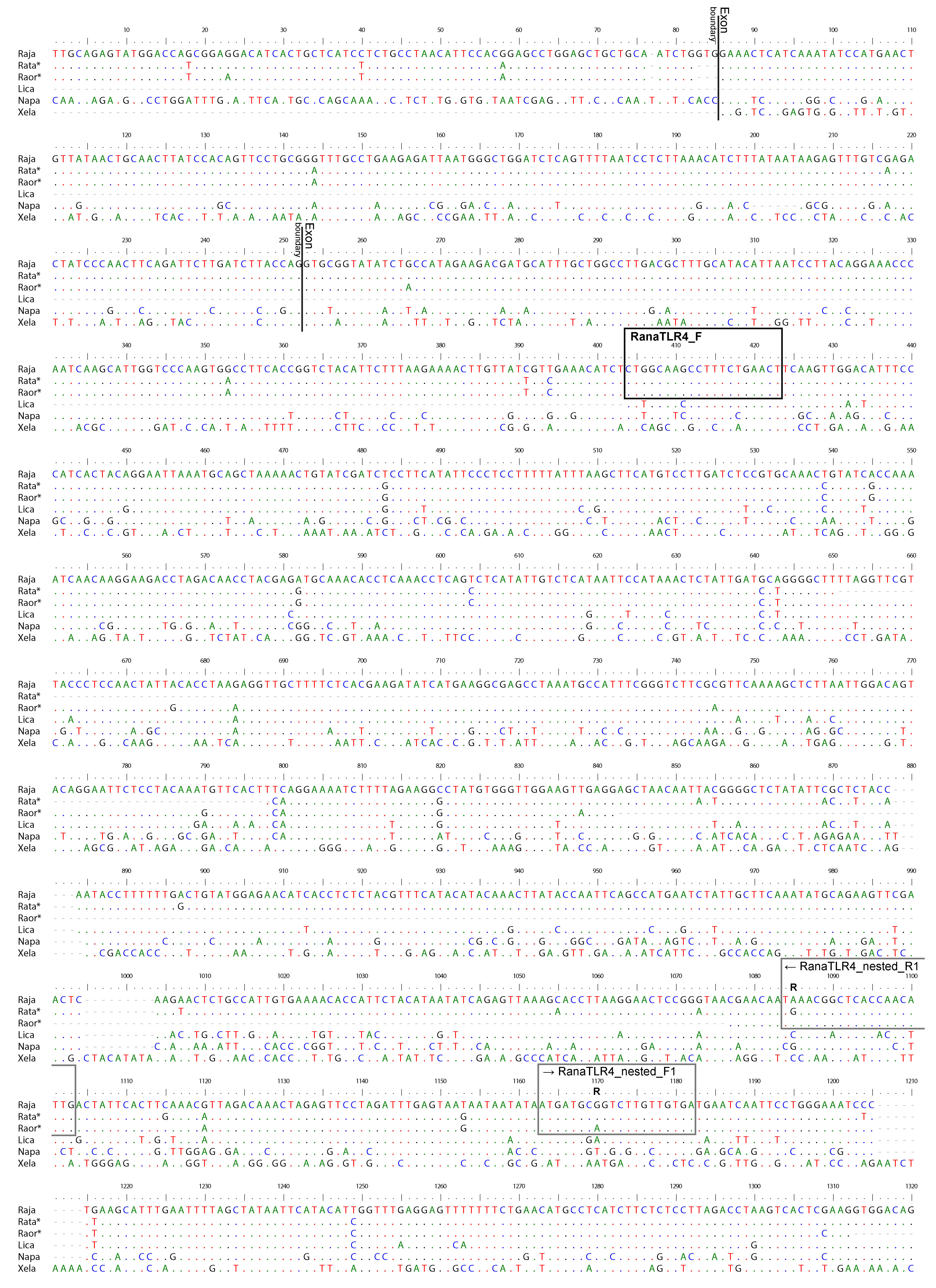

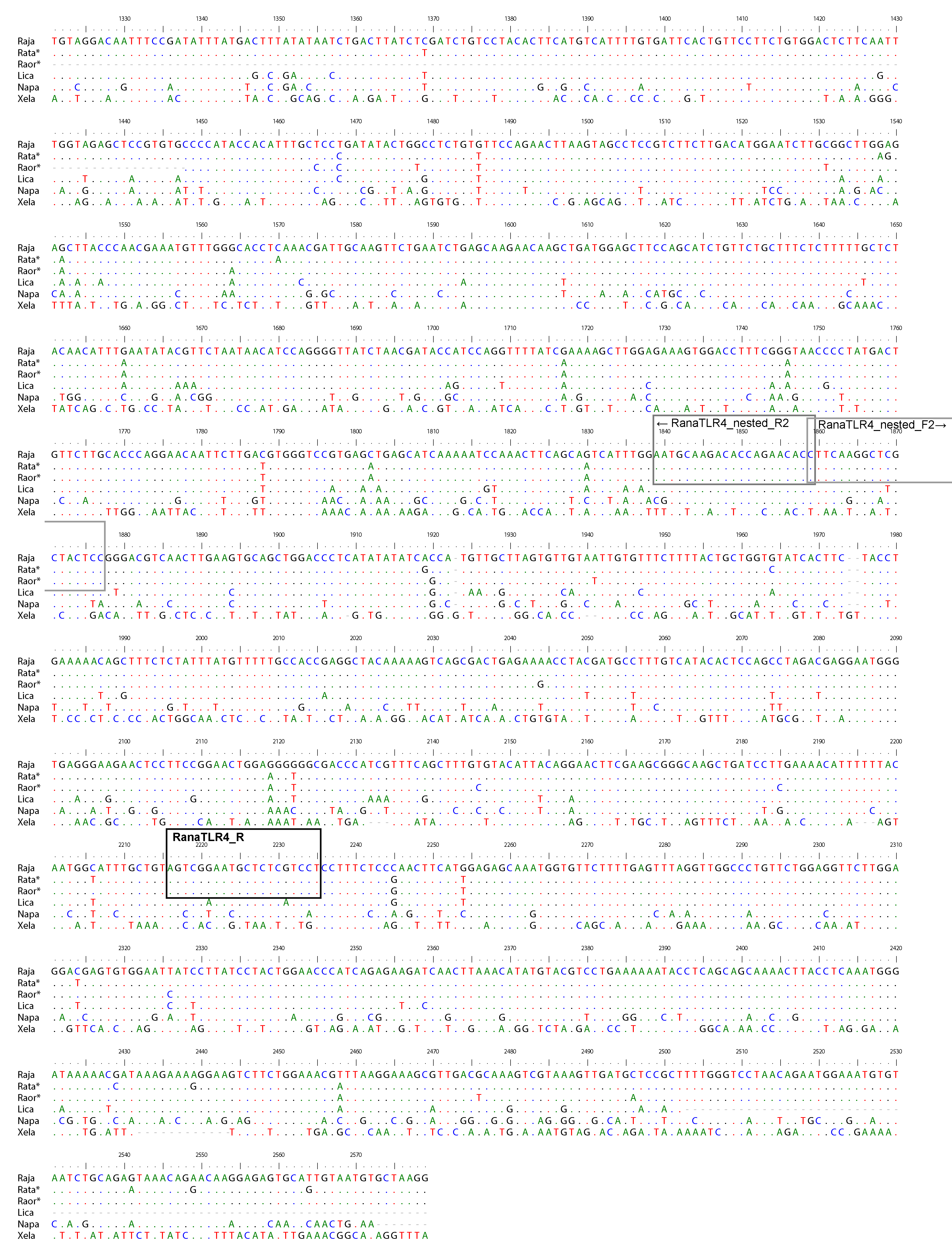


Figure S3. Amino acid alignment of the 27 TLR2 alleles isolated from the three focal species along with sequences from *L. catesbieanus* (Lc, transcriptome data), *X. tropicalis* (Xt, XP_002933537) and *N. parkeri* (Np, XM_018557931). Positively selected sites are indicated by black box. * represents predicted ligand binding sites in human TLR2 (Jin et al 2007). Predicted domains (see Figure S5) are shaded in light green (LRR: leucine-rich repeat), blue (transmembrane region), or dark green (TIR: Toll - interleukin 1 – resistance domain).


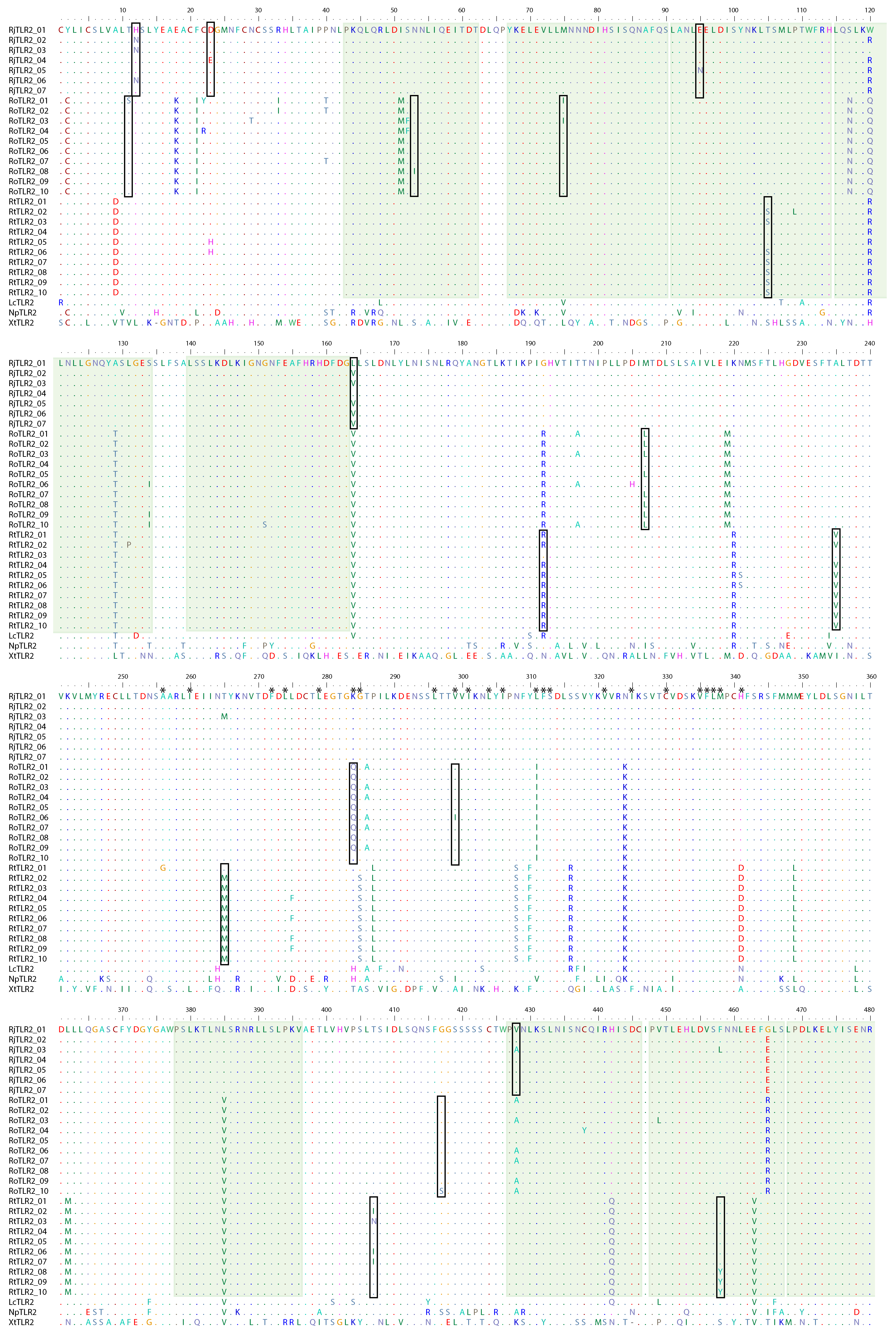


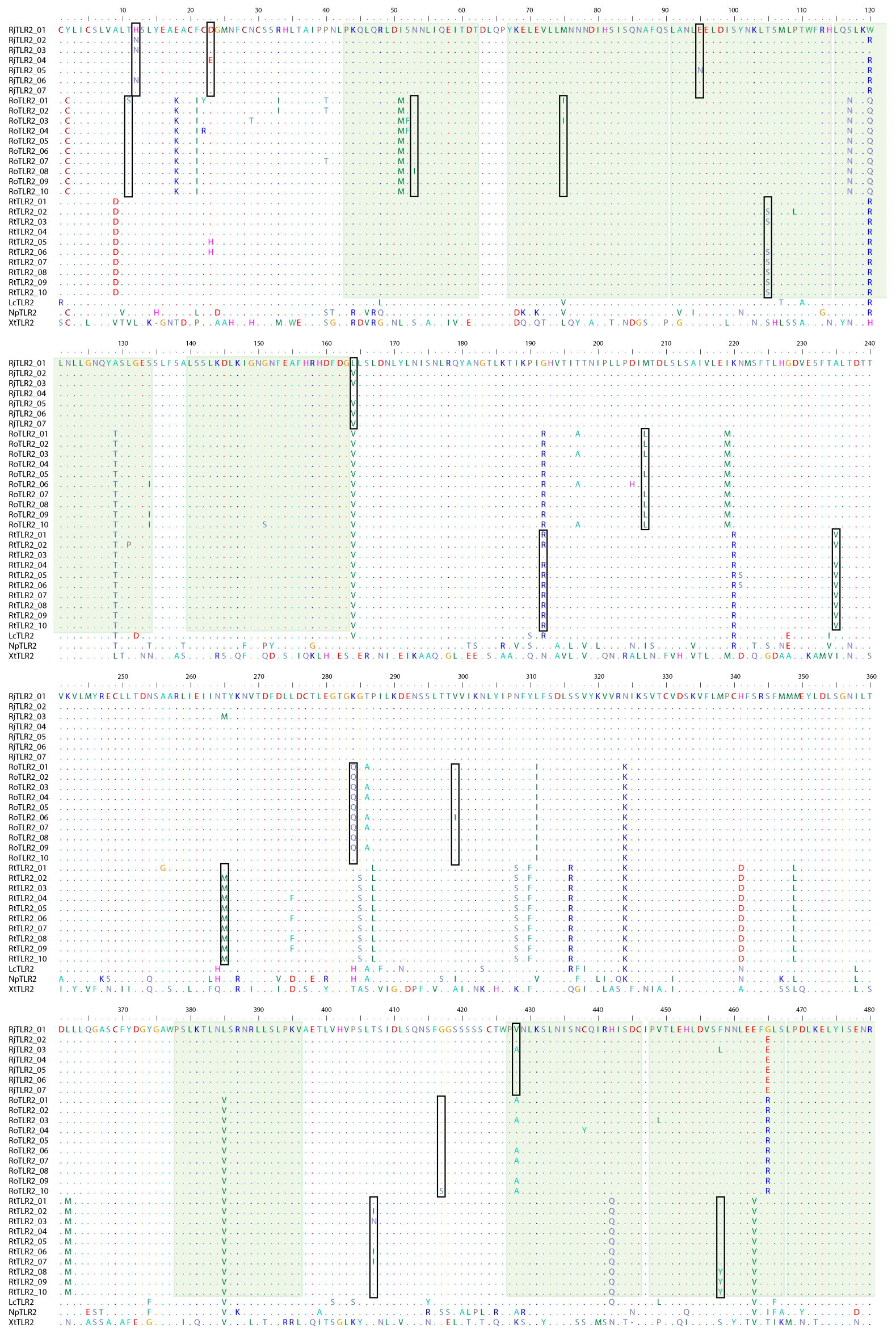


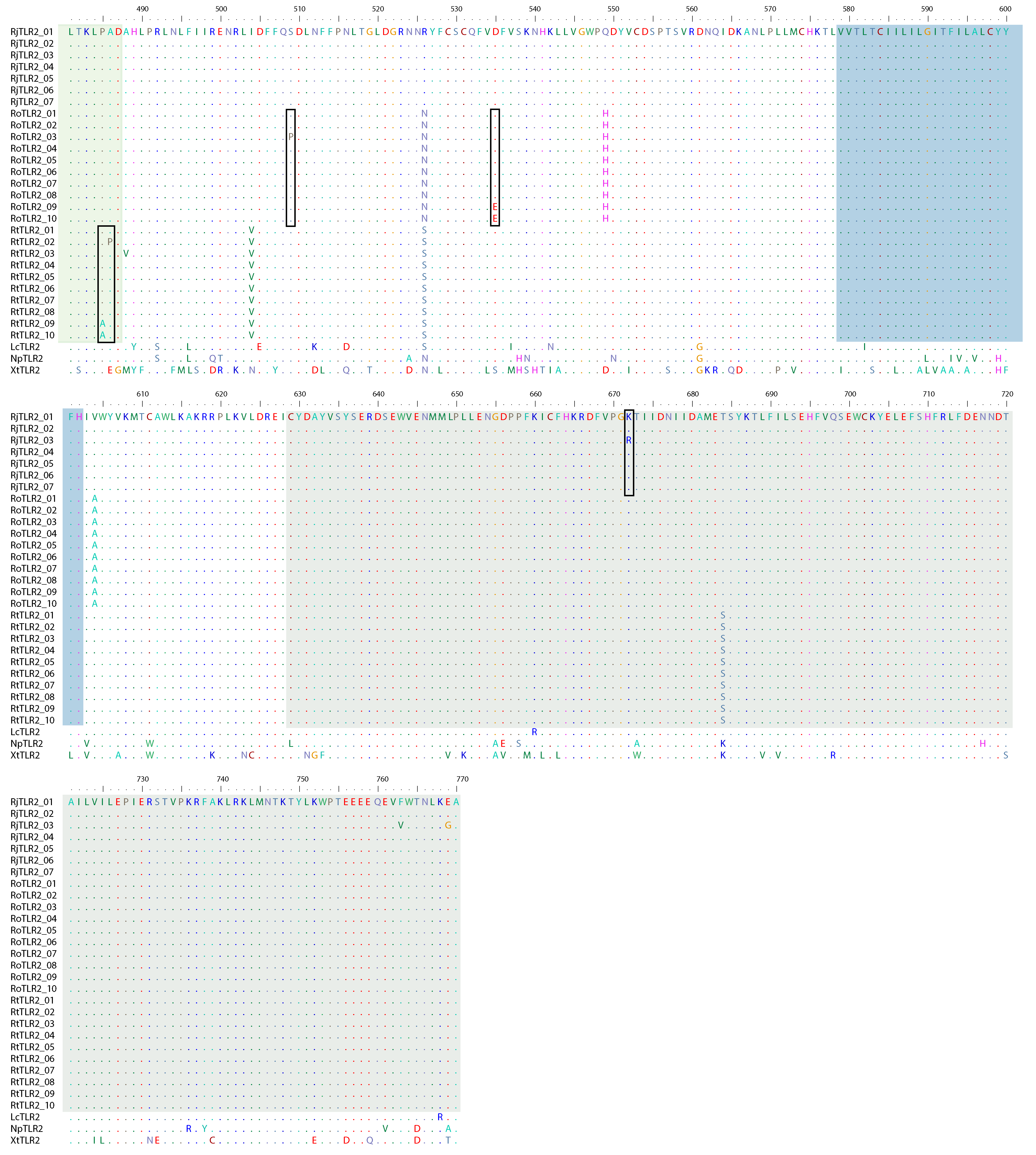


Figure S4. Amino acid alignment of the 20 TLR4 alleles isolated from the three focal species along with sequences from *L.s catesbieanus* (Lc, PIO23183) and *N. parkeri* (Np, XM_018565865). Positively selected sites are indicated by black box. * represents predicted inner core (blue), phosphate (green) or metal/secondary (orange) interaction sites in human TLR4 (Park et al 2009). Predicted domains (see Figure S5) are shaded in light green (LRR: leucine-rich repeat), blue (transmembrane region), or dark green (TIR: Toll - interleukin 1 – resistance domain).


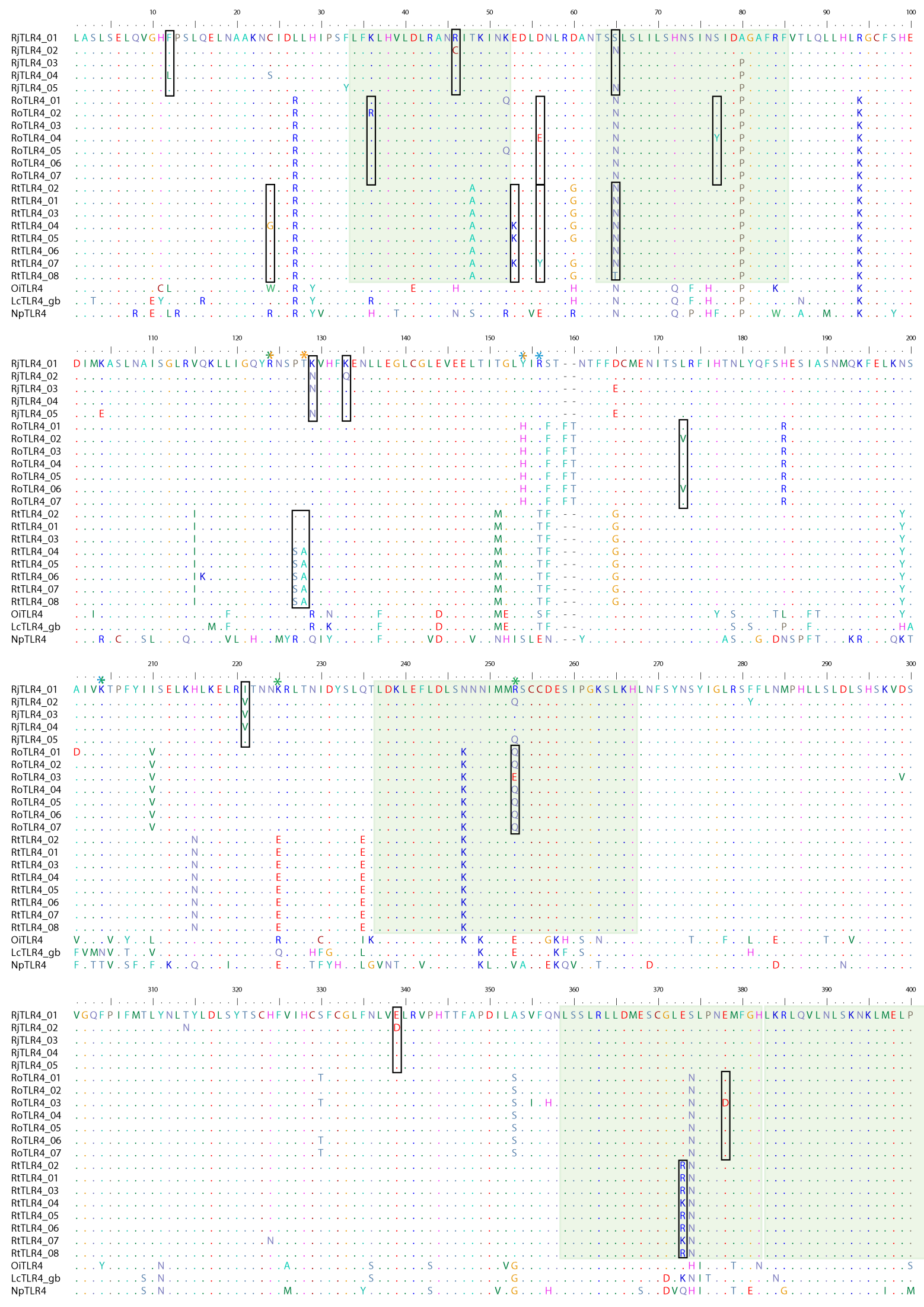


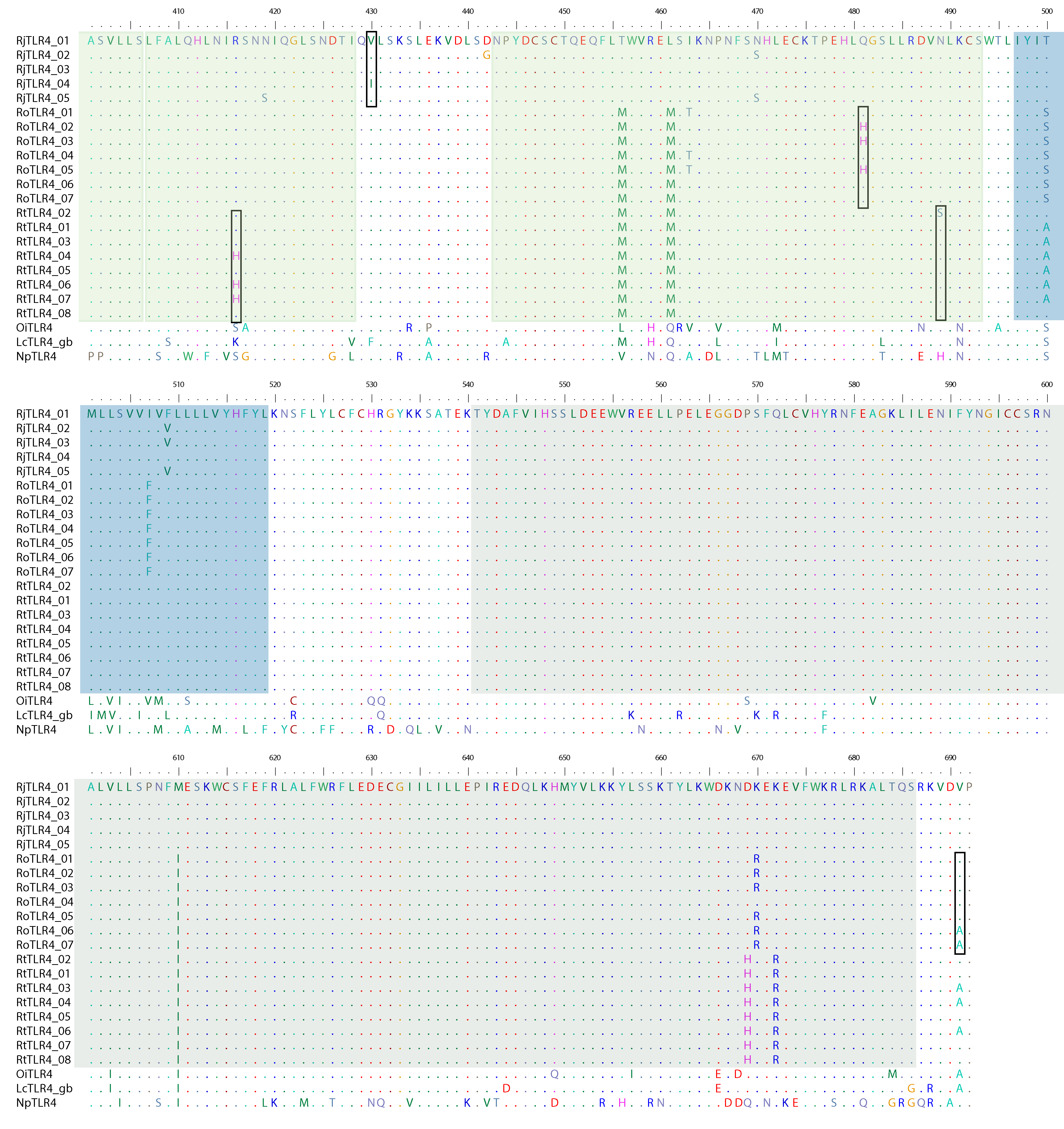


Figure S5. Structure of TLR2 and TLR4 from the three focal Rana species compared to *X. laevis* and *N. parkeri*. LRR: leucine-rich repeat; LRR_CT: LRR C-terminal; TIR: Toll - interleukin 1 – resistance domain. Blue rectangle represents transmembrane region. Domains predicted by the SMART program (http://smart.embl-heidelberg.de).


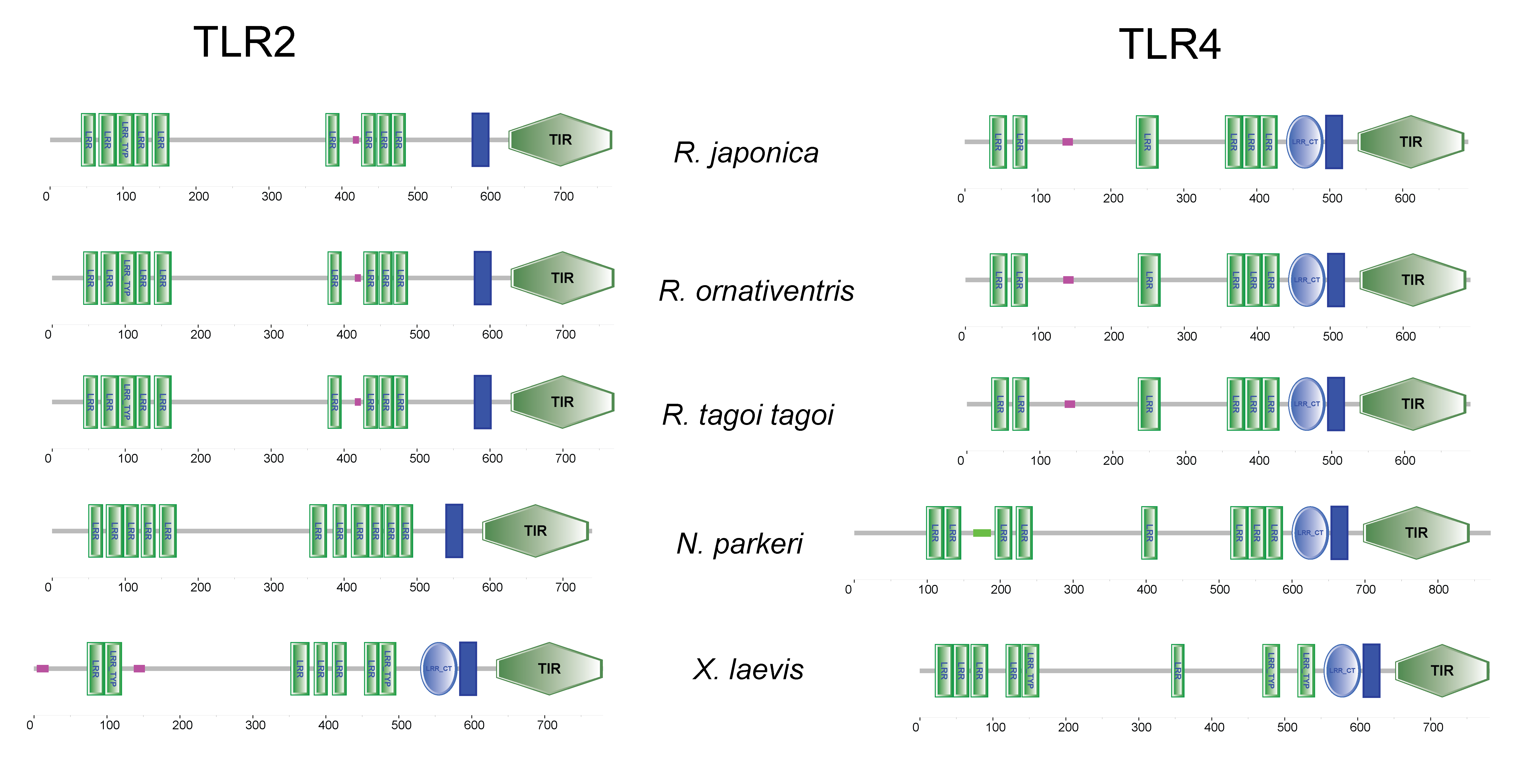

Supplement: Supplemental Information 1 [file peerj-06-4842-s001.docx]
